# Salinity impacts on humidification dehumidification (HDH) desalination systems: review

**DOI:** 10.1007/s11356-023-31327-5

**Published:** 2023-12-13

**Authors:** Ibrahim Nabil, Abdalla M. Abdalla, Tamer M. Mansour, Ali I. Shehata, Mohamed M Khairat Dawood

**Affiliations:** 1 Faculty of Engineering, Mechanical Engineering Dept, Suez Canal University, Ismailia, 44521 Egypt; 2 Mechanical Engineering Department, Arab Academy for Science, Technology and Maritime Transport, Alexandria, Egypt; 3 Present Address: Damanhour univeristy, Faculty of Engineering, Elbehira, Egypt

**Keywords:** Desalination, Salinity, Brine, Humidification–dehumidification, Water productivity

## Abstract

The use of humidification-dehumidification water desalination technology has been shown to be a practical means of meeting the demand for freshwater. The aim of this review is to investigate the impact of salinity on HDH techniques that have various benefits in terms of both economics and the environment, including the capacity to operate at low temperatures, utilize sustainable energy sources, the need for low maintenance, and straightforward construction requirements. Also, in this review, it is observed that the HDH system’s components are strong and capable of treating severely salinized water. It can treat water in an appropriate way than other desalination technologies. This technology has recently been commercialized to treat highly salinized generated water. However, more research is needed to determine how salinity affects HDH productivity. According to several research investigations, while the specific thermal energy consumption increased considerably and the productivity of water per unit of time decreased significantly as the salt mass percentage grew, the purity of clean water did not suffer. The rejected brine must be reduced by increasing the total water recovery ratio in the HDH system. Through this review, it was found that brine control is becoming increasingly important in the water processing industry. ZLD systems, which aim to recover both freshwater and solid salts, can be a viable replacement for disposal methods. Finally, through this reviewer, it was concluded that HDH desalination systems may operate with extremely saline water while increasing salinity has a significant influence on system performance.

## Introduction

The lack of fresh water is one of the most significant issues many communities confront today. It is a global problem that is exacerbated by rapid urbanization, population growth, and economic expansion (Lawal et al. 2018; Elbassoussi et al. 2021a, b). By 2030, it is expected that global water supplies will be around 40% less than what the globe needs. In order to provide the majority of the world’s population with the clean water they require, efficient and affordable small-scale water desalination devices could be developed (Elbassoussi et al. 2021a, b). In the past, several desalination technologies have been created. Solar stills are the most traditional and cost-effective way to get pure water. The water desalination processes are primarily classified into thermal and membrane where thermal desalination is associated with the phase change and the membrane processes are accompanied by a single phase (Ghalavand Y et al. 2015). In thermal desalination systems, the phase change of seawater is implemented using available thermal energy that can be obtained from conventional nonrenewable energy resources like fossil fuel and nuclear or from renewable energy resources such as geothermal energy or solar energy. Solar stills technology, despite not being the most effective, has been the main focus of many researchers due to its ease of use and benefits in terms of cost. New technologies for small-scale water desalination have undergone extensive research and development, and numerous prototypes have been created to test these fresh concepts. Although these prototypes might not be the best option to compete with the existing conventional desalination technologies, they are helpful in the development of dependable, efficient, and affordable decentralized small-scale water desalination systems (Lawal and Qasem 2020).

Recent years have seen a lot of interest in HDH desalination technology. The method offers several appealing characteristics, including the capacity to operate at low temperatures and utilize sustainable energy sources, such as solar and geothermal, and modest technological requirements. The outstanding advantages of HDH desalination systems have prompted numerous theoretical and practical studies (Mohamed et al. 2021).

The essential HDH system parts include a humidifier, a dehumidifier, a water or air heater, pumps, and pipework. Researchers have proposed and examined numerous designs that are formed with various components and cycles. The HDH cycle mimics the cycle of rainfall in nature. It consists of a steam heater and two additional heat- and mass-exchange components, a dehumidifier. and a humidifier. The classification of HDH cycles into water-heated cycles and air-heated cycles based on the kind of heating process is prevalent in the literature (Mohamed et al. 2021). Additionally, the water and air loops in the cycle are primarily divided into closed water open air (CWOA), closed air open water (CAOW), and open air open water (OAOW) systems (Elbassoussi et al. 2021a, b).

Humidification dehumidification (HDH) systems can be used independently or in conjunction with other thermal systems to increase performance. Aiming to improving the effectiveness of increasing the amount of produced freshwater, cooling effect, and power of the combined cycle, special emphasis has been paid to coupling the HDH cycles with refrigeration, power, and other desalination technologies. A possible alternative method to conserve energy and maximize energy use and saving is one that combines an absorption cooling device with a humidification-dehumidification desalination component (Alkhulaifi et al. 2021; Bhowmick and Kundu 2021; McGovern et al. 2013; Nayar et al. 2016). This combination is a creative idea to develop a new thermodynamic system for enhancing the system performance and productivity, among other waste heat recovery methods (Bhowmick and Kundu 2021).

The open-water cycle was mentioned as the primary research topic. Because there is a restricted concentration of salt water in an open water HDH system, salinity has little effect (McGovern et al. 2013). Over the last few years, closed-water cycle systems have drawn increased interest due to the advancement of HDH technology. In order to optimize system configuration, more researchers are starting to use the closed-water cycle. The closed-water cycle, however, can cause the system to become salty. In this situation, the system’s salinity should be adjusted and can be either high or low. The thermophysical characteristics of the saline water are impacted by salinity. For a salinity of 10%, the saturated vapour pressure and specified heat capacity of saline water decline by 6.4 and 11.5%, respectively (Nayar et al. 2016).

According to certain studies, increasing the mass fraction of salt will decrease the humidification efficiency of the system, which will decrease the amount of water the system produces per unit of time (Qing-teng et al. 2019). This suggests that salinity has a substantial impact on the performance of the HDH system.

Therefore, the issues caused by the buildup of salt and the control of salinity have not been addressed up until now, where the performance of the system may differ significantly as a result. This emphasizes the requirement and significance of examining salinity’s impact on system performance. But it is still unknown how salinity affects how well a system works. Furthermore, the majority of earlier studies did not include salinity’s impact on HDH (McGovern et al. 2013; Chehayeb et al. 2015).

Along with the drinkable water, desalination plants also create concentrated brine as trash. The desalination plant’s rejected brine is a serious environmental problem. The cost of additional brine management and treatment, which can make up between 5 and 33% of the overall desalination cost, is decreased by having a desalination system with a lower brine outflow. On the other hand, brine disposal into the ocean or into waterways that are related to the ocean can have serious negative effects on the environment (Elmutasim et al. 2018). It may result in several issues for both the sea environment and the subsurface habitat. A particularly beneficial technique to lower the expense of brine management in subsequent steps is to use a brine minimization strategy prior to rejecting the brine out of the desalination system. Brine treatment is one of the most promising options to brine disposal since it reduces environmental pollution, minimizes waste volume, and produces freshwater with a high recovery rate (Panagopoulos et al. 2019).

The HDH desalination process was reviewed in this study since it is a recently commercialized method of treating highly salinized water. Reviewing the HDH water desalination systems with varying salinities will be the main emphasis in order to evaluate the effects of salinity on water productivity and system performance, as well as the relationship between salinity and other operational parameters and how it affects overall cost. It also discusses the properties of brine, how it affects the environment, and the most effective methods employed in brine treatment for desalination. One of the most effective experimental techniques for reusing industrial wastewater and reducing the harm done to the environment by brines from desalination systems is to use zero liquid discharge systems. The humidification-dehumidification systems may be a suitable option for zero liquid discharge applications.

## Methodology

After defining our research objectives, we started searching the literature, and we selected the databases to find the most relevant studies of salinity impacts on humidification dehumidification (HDH) desalination systems. The databases chosen were Scopus, Science Direct, and EBSCOhost. In the next phase, the selection of keywords was made. Desalination, salinity, brine, humidification–dehumidification, and water productivity were chosen to obtain results according to the topic and thus reduce the selection biases. After that, the time interval was defined. The selected period was from 1980 to 2022 to analyze the impact of salinity on HDH water desalination system. The following step focused on setting the inclusion and exclusion criteria; we include only journal articles and conference papers and exclude thesis, books, and manuals. We only include papers that were written in English and remove those in other language. We proceeded to read the summaries, to reduce redundancies and repetition and considering only the relevant documents. Finally, a manual search of other documents was made using complementary databases: Research Gate and Google Scholar. As a result, relevant articles were admitted carrying out the review.

## Humidification–dehumidification desalination system

**Fig. 1:**
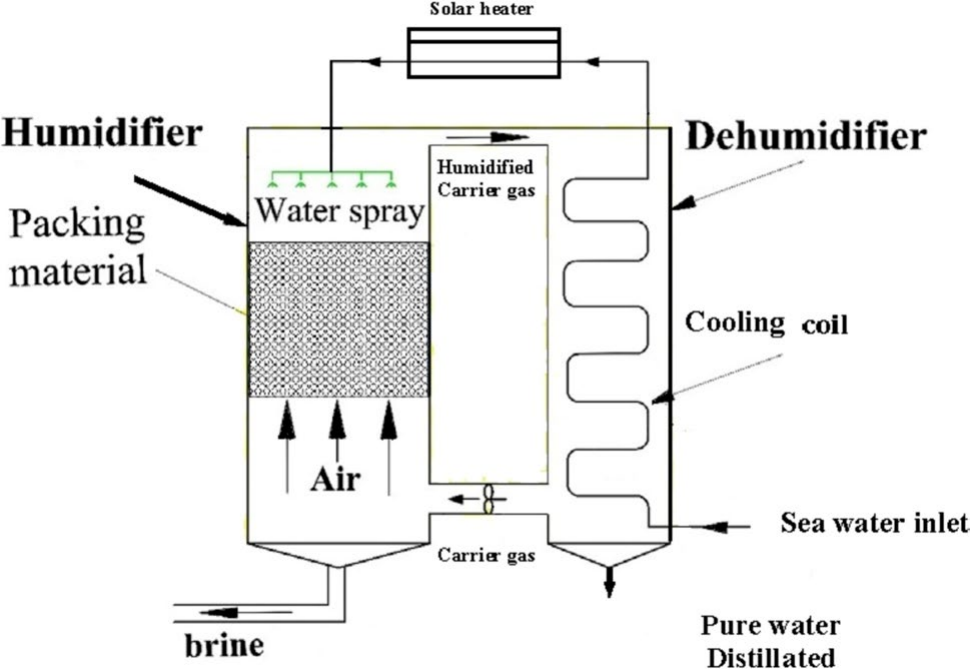
Schematic of a simple humidification-dehumidification process

The HDH desalination main principle is that saltwater is converted to water vapor in a humidifier, where it condenses over condensing coils in a dehumidifier at a surface temperature that is lower than the dew point of ambient air. The evaporation process is accelerated by heating the water, the air, or both to increase the air’s capacity to carry more water vapor and so boost the productivity of freshwater HDH WDS. Theoretically, a temperature increase from 30 to 80 °C can cause 1 kg of dry air to convey a half kilogram of vapor with an energy usage of 2814 kJ (Kabeel et al. 2013). During the humidification process, moving air that is kept in contact with seawater removes a certain amount of water vapor. When humid air comes into touch with a cold surface during the dehumidification process, water vapor condenses to provide the necessary fresh water. The latent heat of condensation is used during the condensation process to preheat seawater as it travels through the condensing coils (Kabeel et al. 2013). The cycle of the seawater can be either an open cycle or a closed cycle in the HDH desalination process. A reduction of 60 °C for 10 kg of seawater is caused by 1 kg of water evaporating in the open cycle. Between 5 and 20% of the seawater that is circulated in the system is made up of fresh water. With a considerable heat loss, this translates to a limited freshwater yield. With less energy used in a closed cycle, greater freshwater output can be maintained (Moumouh et al. 2018). Figure 1 shows a simplified example diagram for the humidification-dehumidification (HDH) water desalination process.

It has been noted that the HDH system essentially comprises two vertical ducts joined at the top to circulate air. The HDHWDS desalination system can be run in forced draft or natural draft modes, depending on the airflow. The condenser, which creates the unsaturated air, is included in one duct that represents the dehumidifier. An evaporator delivering saturated air is a packing material built into the other duct to effectively humidify air. The air and/or water are heated utilizing a variety of energy sources, such as thermal, solar, geothermal, and hybrid, to improve system efficiency.

The humidifier (evaporator), which evaporates the water sprayed on top of packing materials as shown in Fig. 1, is the basic component HDH distillation system. The dehumidifier (condenser), on the other hand, condenses the humidifier’s produced incoming vapor. As can be seen from the illustration, the condensate water is collected from the condenser bottom. In addition, the produced vapor is transferred from the humidifier to the dehumidifier using air, which is regarded as the HDH system’s carrier gas. In order to increase the efficiency of evaporation, the researchers heated the water and the air while maintaining the same ambient pressure. The bottom of the humidifier and dehumidifier sections yields the HDH system’s final results, which are freshwater and brine-rejected water, respectively (Mohamed and El-Minshawy 2009). Regarding the media heating of the distillation unit, HDH systems are divided into three categories: water-heated, air-heated, and dual heated. In order to improve the evaporator’s efficiency, it was already noted that the air and water were heated. In HDH systems that are air-heated, the system is powered by heat sources and humid air. The HDH unit is operated by a water-heated system that is created by heating saltwater. Use of the dual heated system results in increased efficacy and freshwater output (heating the humid air and water). The two types of HDH distillation units’ direct and indirect water heating are categorized in accordance with the external energy source used to heat the water. When solar radiation is directly absorbed by the feed water used as input by the desalination plant, it is considered to be of the direct kind. Solar radiation is captured by thermal collectors, which either transfer the energy to the salty water in indirect plants or transform it into electricity that is subsequently used to power the plant. In both cases, a heat engine could be used to power the plant using energy (Ghazouani, et al. 2022).

The evaporator, condenser, and heat exchanger are the three parts that make up the air-heated kind of HDH. The advantage of closed-air and open-water (CAOW) units is that they continuously distil freshwater. The evaporator experiences simultaneous heat and mass transmission. The seawater in the humidifier evaporates due to the pressure differential between the humid air and the preheated water that is coming from the dehumidifier. As a result, air humidity and temperature rise. While the evaporator’s bottom is being emptied of its residual seawater and its temperature reduced. Additionally, the condenser’s seawater inlet cools the humidifier’s created vapor before it is used to produce freshwater. Once the humid air has been released, the air cycle within the distillation unit is shut off. In this distillation machine, it is also possible to extract the latent heat energy from humid air (Mohamed and El-Minshawy 2009; Yuan et al. 2011).

## Performance parameters of HDH system

The GOR gain output ratio, specific water production, recovery ratio (RR), mass flow rate ratio (MR), energy reuse factor (*F*), and specific entropy generation (${S}_{\mathrm{gen}}$) are some of the indicating metrics used to evaluate the effectiveness of the HDH WDS.

### Gained output ratio

One of the primary performance measures for the HDH WDS is the gained output ratio (GOR). It is defined as the ratio of the latent heat of vaporization of the produced fresh water to the total energy input to HDH WDS, and it can be computed using the following formula (Mistry et al. 2011):

$$\mathrm{GOR}=\frac{{m}_{fw}{\prime}\lambda }{{Q}_{in}{\prime}}$$

where ${m}_{fw}{\prime}$ the mass flow rate of freshwater, *λ* is the latent heat of vaporization, and ${Q}_{in}{\prime}$ is the total energy input. Low heat input is sought per unit mass of fresh water, which is indicated by a high GOR value. As a result, utilizing fossil fuel as a heat source and obtaining a higher GOR value equate to cheaper fuel expenditures. The beginning expenses are lower when energy is used as a heat input resource since a smaller solar collector is needed to maintain a greater GOR (Kabeel et al. 2013).

### Specific water production

The specific water production is the amount of fresh water gained daily per square meter of the collector area. Simply, this parameter is used to examine the HDH WDS’s solar energy utilization efficiency. In addition, the HDH WDS capital cost evaluation is necessary because the cost of solar collectors for the air heating system ranges from 40 to 45% (Kabeel et al. 2013; Narayan et al. 2010) Along with the water heating system, which ranges from 20 to 35% (Narayan et al. 2010).

### Recovery ratio

The recovery ratio (RR), which is measured as the quantity of fresh water per kg of feed saltwater, is greatly improved as a result of improving the salt rejection. When saltwater was desalinated with a salinity of 35,000 mg/L in the 1980s, the RR was approximately 25%; however, by the 1990s, it had increased to almost 35%. A system with a second stage can achieve a RR of 60%, which is currently about 45% (Ghaffour et al. 2013). This is determined using (Sharqawy et al. 2014a, b).

$$\mathrm{PR}=\frac{{m}_{fw}{\prime}}{{m}_{sw}{\prime}}$$

where the mass flow rate of feed seawater is ${m}_{sw}{\prime}$.

## Thermophysical properties of seawater

Dissolved salts alter the thermophysical characteristics of water, which can make the operation of HDH systems dependent on the feed’s salinity. McGovern et al. (2013) have demonstrated that employing pure water attributes results in calculation errors of no more than 4–5% for feeds at oceanic salinities or below. Water produced during oil and gas extraction may have substantially greater salinities than those seen in brine concentration. Most seawater’s physical characteristics are comparable to those of pure water regarding pressure and temperature. The mass of dissolved salts per unit mass of salt water, which is defined as salinity, should be recognized as a third independent variable in addition to temperature and pressure since seawater is a mixture of pure water and sea salts. Density, specific heat capacity, and boiling point elevation are only a few examples of parameters whose fluctuation significantly influences the efficiency of distillation systems, even though the differences between pure water and sea water are just in the range of 5 to 10%. In order to model, analyze, and develop various desalination processes, it is required to precisely define the physical and thermal characteristics of seawater. Many experimental observations have been made using “synthetic” saltwater that has been made by combining the right salts with distilled water and occasionally leaving out calcium sulfate because it would be needed for evaporation at higher temper0atures (Fabuss 1980). Some measurements, though, have been made using pure seawater. In this regard, samples of saltwater with higher salinities were concentrated through evaporation, whereas samples with lower salinities were created through dilution. The temperature and salinity of the brine in desalination systems may also reach levels that are significantly greater than the oceanographic range (*S* = 0–40 g/kg and *t* = 0–40 °C). For instance, depending on the specific technology, the top brine temperature in thermal desalination systems is normally between 60 °C and 120 °C. However, a brine discharge can be anticipated to have a salinity that is between 1.5 and two times greater than the feed saltwater in typical seawater reverse osmosis (SWRO) systems. Therefore, the ranges of 0–120 °C and 0–120 g/kg in salinity are of importance for desalination operations (Sharqawy et al. 2010).

### The relation of seawater specific heat variations with temperature and salinity

**Fig. 2:**
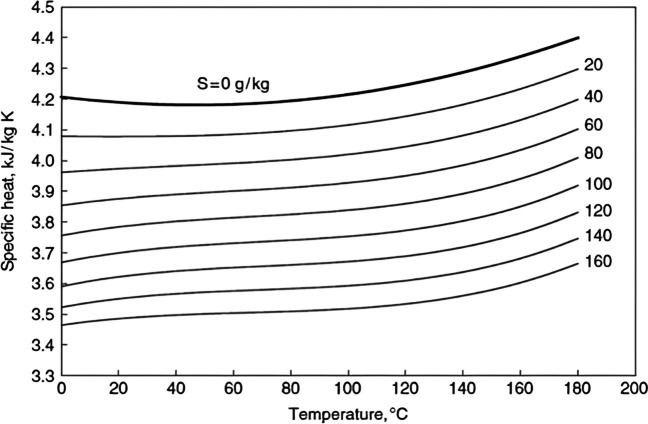
Seawater specific heat variations with temperature and salinity calculated using (Fabuss 1980)

Numerous researchers have measured the specific heat of saltwater (Millero et al. 1973), and it is available over a large range of salinity (0–120 g/kg) and temperature (0–200 °C) (Fig. 2).

### Latent heat of vaporization

**Fig. 3:**
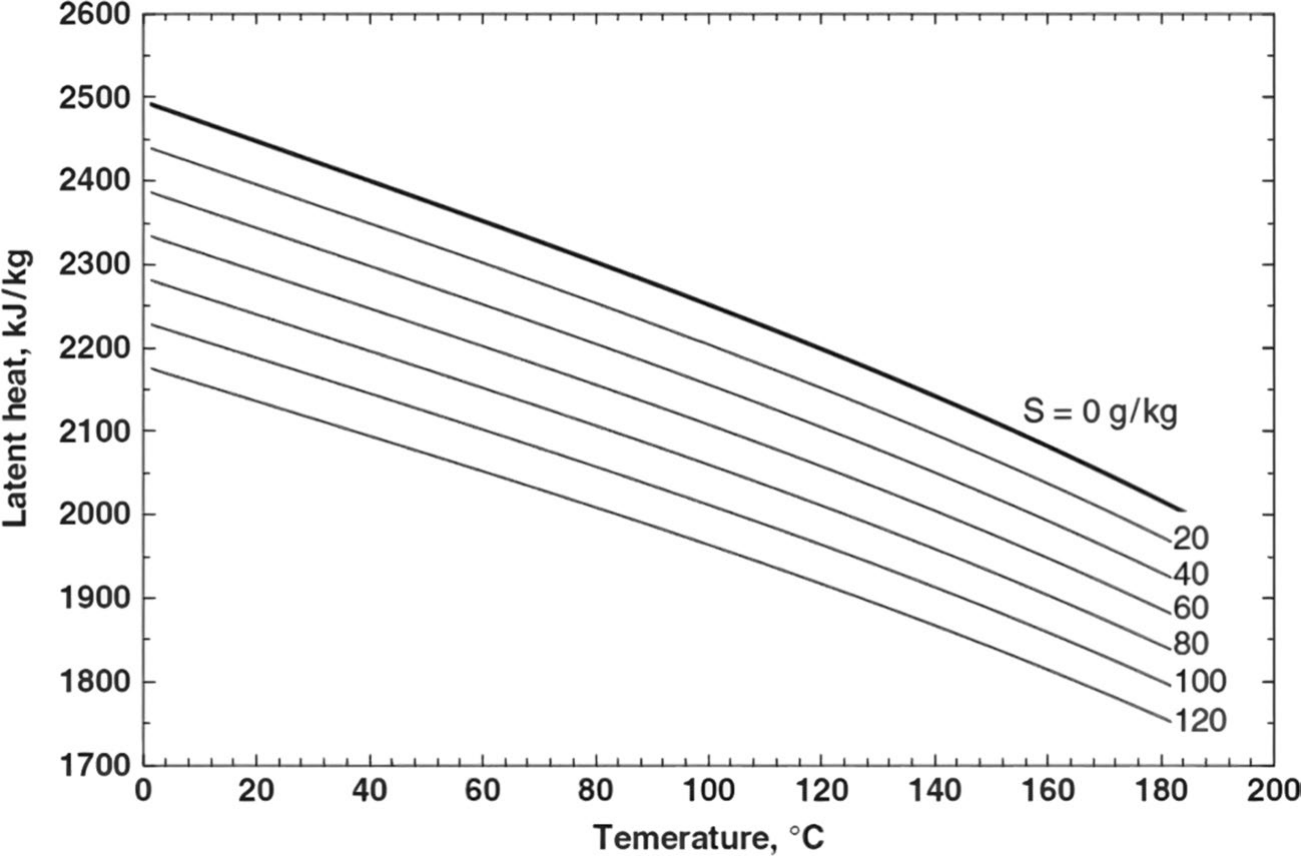
Seawater latent heat variations with temperature and salinity (Fabuss 1980)

The latent heat of vaporization is the quantity of heat needed to change a unit mass from a liquid to a gaseous state. The temperature affects the latent heat of vaporization for pure water. For the change of latent heat with salinity and temperature in saltwater, no formulas appear to be available (Sündermann and Börnstein 1986) (Fig. 3).

## Effect of feed water salinity on the performance of HDH system

A thermal desalination technique called humidification dehumidification (HDH) simulates the rain cycle in a designed environment. Small-scale, decentralized applications might benefit from it. It can also clean very salty water. Recently, the method for treating highly salinized produced water from hydraulically fractured oil and gas wells became commercially available. That facility has demonstrated HDH’s capacity to treat water that most contemporary saltwater desalination systems are unable to treat (Pankratz 2014). 

Brine recirculation is a realistic approach to reduce the volume of rejected brine in the HDH system because the humidification process can occur at high salinity feed. As opposed to indirect dehumidification, direct contact dehumidification completely removes the corrosion-related problems associated with excessive feed water salinity. The overall recovery ratio of the system improves when the salinity of the recirculating brine is increased from 10 to 30% (from around 66 to 86%). The system’s GOR, however, drops marginally from roughly 0.65 to 0.45 (Dehghani et al. 2019).

With various salinity levels, Huang et al. (2020) used a closed-water multi-stage HDH desalination system. For every 1% rise in salinity, water productivity decreases by 0.75%. Maximum energy efficiency is also affected (in an average of 11.44% for 20% salinity). To keep the system’s maximum energy efficiency higher than that of an open-water system, it is recommended that the salinity level be kept below 15%. In order to evaluate the system performance at various salinity levels, the parameter known as the pinch point heat capacity rate ratio is also introduced. When the first-stage dehumidifier’s pinch point heat capacity rate ratio is equal to unity at any salinity level, energy efficiency is maximized. Meanwhile, depending on whether the pinch point heat capacity rate ratio of the first-stage dehumidifier is less than or higher than unity, it is possible to determine whether salinity plays an enhanced or lessened role in energy efficiency.

According tov Qing-teng et al. (2019) study on the treatment and concentration of high salinity water using the HDH process, the results showed that as the mass fraction of salt increased, the productivity of water per unit of time decreased significantly, the consumption of specific thermal energy increased significantly, the quality of pure water remained unaffected, and the salt rejection ratios were maintained above 99.9%.

Dehghani et al. (2019) investigated a method of brine recirculation that uses a bleeding flow of concentrated brine stream. The system can operate at steady state with a high salinity input thanks to this. To test the applicability of the approach, experiments are carried out for feed salinities in the range of 10–30%. To assess the practicality and thermal performance of the suggested brine recirculation approach, an open-air water-heated HDH desalination system with a direct contact dehumidifier is put through its paces. Based on the results, it is possible to implement the suggested brine recirculation and achieve a general recovery ratio of around 88% at a salinity of 30%. By reducing the volume of rejected brine, this technique can greatly ease problems with HDH desalination systems’ brine management.

In an experimental comparison of a single-stage and three-stage vacuum humidification-dehumidification (VHDH) system at sub-atmospheric pressure conditions, Rahimi et al. (2020) found that the salinity change had no discernible impact on the performance of the MS-VHDH system. The findings indicated that the desalination rate was typically reduced by around 4% as a result of salinity change.

Thiel et al. (2015) investigated how high salinities affected the efficiency and effectiveness of an HDH system with a brine recirculation option. According to the findings, the zero extraction HDH desalination system uses about 370 kWh/m^3^ of energy. The GOR of the HDH system was also largely unaffected by the feed salinity.

El-Agouz and Abugderah (2008) discovered that the system’s ability to produce water per unit of time will decrease as the mass percent of salt increases.

Chen et al. (2018) demonstrated that a spray-assisted low-temperature desalination system with a feed flow rate of 3.8 LPM, a cooling water flow rate of 3.8 LPM, and a solar collector area of 7.6 m^2^ can deliver a continuous supply of fresh water of 30 L/day. A tiny amount of the hot brine is recycled to increase thermal efficiency and boost productivity, and the output rate is inversely related to the feed salinity.

Chehayeb and Lienhard (2015) studied a humidification dehumidification system with a variable feed salinity and found that, even at high salinity, it is advantageous to apply a modification in the mass flow rate ratio by injection/extraction. Because the humidifier allows air to get supersaturated without being constrained by the saturation water content, the detrimental effects of growing salinity on GOR are lessened. Furthermore, HDH is appropriate for operation under fluctuating feed salinity since the optimal mass flow rate ratio is not a strong function of feed salinity.

Rahimi et al. (2020) studied experimentally and thermodynamically a solar-assisted pilot-scale vacuum humidification-dehumidification (VHDH) desalination system. The findings indicated that increasing the salt of the water will increase its viscosity and surface tension while lowering its latent heat of vaporization. By maintaining the humidifier temperature below 68 °C, which prevents the process’s strong volatility in thermodynamic characteristics, the influence of salinity changes on energy efficiency is reduced. It is shown in the study, “The effect of feed water salinity variation from 10 to 35 g kg^−1^ on exergy efficiency at *P* = 70 kPa,” that switching from brackish to seawater salinity causes only a small change in productivity, absolute humidity, and temperature, but not a significant difference in exergy efficiency. The energy efficiency is poorer at 35 g kg^−1^ salinity than it is at 10 g kg^−1^, as would be predicted. It results from the slight modification of heat and mass transport in the humidifier and the dehumidifier at the two salinities indicated.

Experimental research was done by Shalaby et al. (2021), who used a hybrid solar humidification-dehumidification system. The suggested desalination system was found to provide fresh water with a TDS range of 138 to 148 ppm, regardless of the salinity of the feed water. The main attractive factor in using the HSHDH for desalinating highly salinized water is that the salinity of the water has no impact on the production of freshwater. Using high salinity water with a TDS of 100,000 ppm instead of brackish water with a TDS of 1660 ppm resulted in a reduction of the overall electrical energy consumption by 65%. When utilized for desalinating high salinity water, this gives HSHDH additional benefits than other desalination systems like MD or RO since the water’s salinity has no effect on its productivity. Additionally, high salinity water was used with very little electrical energy use.

According to the Thiel et al. (2015) study, generated water has been used to explore the impact of increased salinity on desalination system performance. The minimum amount of effort needed to desalinate generated water depends on the salinity but can be up to 9 kWh/m^3^, which is nearly an order of magnitude more than the general rule of thumb for the least amount of work needed to desalinate seawater at 50% recovery. The GOR varies from 1 to 3, just like for zero-extraction HDH, depending on the system size and brine salinity. The GOR of HDH with brine recirculation is often independent of feed salinity. Performance may be enhanced by using different extraction and injection setups. For the HDH system, efficiency models were built. We can infer the following information about the system’s performance using a fixed brine salinity of 26%.

### Effect of feed salinity on the performance of HDH systems

Most of the research that investigated the effect of feed salinity on water productivity was summarized in Table 1.

**Table 1:** Salinity level of feed water in HDH

Author	Study category	System description	Type of feed water	Salinity	Productivity	GOR
Rahimi et al. (2020)	*Experimental*	MS-VHDH	Brackish and saline water	0 − 35 ppt	3.6 kg/h	–
Rahimi et al. (2020)	*Experimentally and theoretically*	VHDH	Brackish and seawater	10–35 ppt	2.4 kg/h	2.50–2.62
Cao et al. (2021)	*Theoretical*	SGSP, KC, HDH	Saline water	35 ppt	152–586 kg/h	1.63
Elbassoussi et al. (2020)	*Mathematical*	Hybrid AD-HDH	Seawater	35 ppt	21.75 kg/h	2.5
Sharqawy et al. (2014a, b)	*Mathematical*	OACW-AH and WH	Saline water	35 ppt	10 kg/h	WH 1.93 AH 2.197
Orfi et al. (2004)	*Experimental and theoretical*	OAOW-AH-HDH-HP	Saline water	2.84 ppt	11.46 kg/h	–
Gabrielli et al. (2019)	*Mathematical*	HDH-PVT	Saline water	20 ppt	0.32 kg/h	–
Lawal et al. (2020a, b)	*Experımental*	HDH-HP	Saline water	2.44 ppt	11.99 kg/h	4.07
Elbassoussi et al. (2021a, b)	*Mathematical*	Hybrid TEAB-HDH	Saline water	35 ppt	10 kg/h	5
Ghiasirad et al. (2021)	*Theoretical*	Multi-generation-HDH	Seawater	35 ppt	3837.5 kg/h	1.406
Elbassoussi et al. (2021a, b)	*Numerical*	Hybrid HDH and ADHP	Saline water	35 ppt	152.4 kg/h	SEAB 10 DEAB 15
Qasem et al. (2020)	*Mathematical*	DEAB-HDH-CAOW	Seawater	35 ppt	1145 kg/h	4.54
Alkhulaifi et al. (2021)	*Mathematical*	ECS-WH-CAOW HDH	Seawater	42 ppt	13.72 kg/h	0.47 and 0.78
Qasem and Zubair (2019)	*Mathematical*	Hybrid HDH and AD	Seawater	180 ppt	20–30 kg/h	7.8 7.6
Lawal et al. (2020a, b)	*Mathematical*	(OWCA) HDH	Saline water	35 ppt	8.5 kg/h	variable
McGovern et al. (2013)	*Mathematical*	Closed air water-heated HDH	Saline water	35 ppt	–	14
Kabeel and El-Said (2013)	*Mathematical*	Hybrid HDH–SSF	*Saline water*	1 ppt	7.42 kg/h	10.2
Zubair et al. (2018)	*Experimental and mathematical*	(OWOA) HDH (CWOA) HDH	Seawater	35 ppt	–	0.4 and 0.42
Elsafi (2017)	*Mathematical*	AH-HDH-CPVT	Seawater	35 ppt	3.66 kg/h	–
Giwa et al. (2016)	*Mathematical*	PV-HDH	Sea water	35 ppt	5.8 kg/h	–
Alrbai et al. (2022)	*Experimental*	CAOW-AH-WH	Seawater	40.6 ppt	0.3804 kg/h	3.4
Shalaby et al. (2021)	*Experimental*	FPSWH and HSHDH	Saline water	1.66 ppt	9 kg/h	–
Abdelaziz et al. (2022)	*Experimental*	HDH and HFUA	Saline water	6 ppt	8.57 kg/h	1.54
Dehghani et al. (2019)	*Experimental*	HDH	Seawater	10–30 ppt	4.9 kg/h	0.65 to 0.45
Huang et al. (2020)	*Mathematical*	MS-HDH	Saline water	3.5, 5, 5.18, 5.39 ppt	63.12 kg/h	2.89 to 3.43
Qing-teng et al. (2019)	*Experimental*	HDH	Saline water	3.5, 10, 15, 20 ppt	1.6 kg/h	0.9
Chehayeb and Lienhard (2015)	*Theoretical*	HDH-MTBC	Saline water	0–200 ppt	–	3.8

## Brine treatment and zero liquid discharge approach

Due to its high salinity, brine, often referred to as a concentrate, is a by-product of the desalination process that has a negative effect on the environment. Brine management methods must be practical and economical in order to lessen environmental pollution. Surface water discharge, sewage discharge, deep well injection, evaporation ponds, and land application are some of the disposal techniques now used. These brine disposal techniques, however, are not viable due to their high capital costs and limited applicability. Since treatment reduces environmental contamination, and waste volume, and produces freshwater with a high recovery rate, it is now thought of as one of the most viable alternatives to brine disposal (Panagopoulos et al. 2019).

Engineers are driven by these pressing needs to create a desalination system that has the best chance of increasing water recovery by lowering brine levels while causing the least amount of environmental harm. Consequently, using a treatment method known as zero liquid discharge (ZLD) will allow for the fulfillment of these requirements. According to what its name implies, ZLD is a combination of desalination methods designed to produce high-quality freshwater while completely eliminating liquid waste from the plant (Alnouri et al. 2017).

### Zero liquid discharge systems based on the HDH process

One of the most effective experimental techniques for reusing industrial wastewater and reducing the harm done to the environment by brines from desalination systems is to use zero liquid discharge systems. Modified humidification-dehumidification systems may be a suitable option for zero liquid discharge applications because of their straightforward fabrication process, low maintenance costs, high inlet water quality requirements, and capacity to use renewable and low-grade heat. In order to operate with minimal liquid discharge, three cutting-edge brine recycle humidification-dehumidification systems are thoroughly explored. The first system runs on heat: (1) A heat-powered brine recycle humidification-dehumidification system; the other two systems are powered by electricity. (2) A brine recycle humidification-dehumidification desalination system connected to a heat pump in which the evaporator acts as the dehumidifier, and (3) a brine recycle humidification-dehumidification desalination system connected to a heat pump in which the evaporator acts as the humidifier (Ghofrani and Moosavi 2020a, b). The concept of ZLD is gaining more and more interest in both the academic and professional worlds. ZLD is a type of treatment that converts brine into solid dry salt and purified water, doing away with the requirement for wastewater disposal. Brine concentrators and crystallizers are the main components of a standard ZLD brine treatment system. The former increases the salinity of the desalination brine (from 70 g/L to more than 200 g/L, for example), and the latter crystallizes the salts to separate them from the liquids. Energy-efficient techniques as MED Panagopoulos or osmotically aided reverse osmosis (OARO) by Bartholomew et al. (2017) and Liang et al. (2021) can be used to concentrate brine. The ZLD system’s bottleneck is the crystallization process, which even though it only recovers a small amount of water uses more energy than brine concentration.

**Fig. 4:**
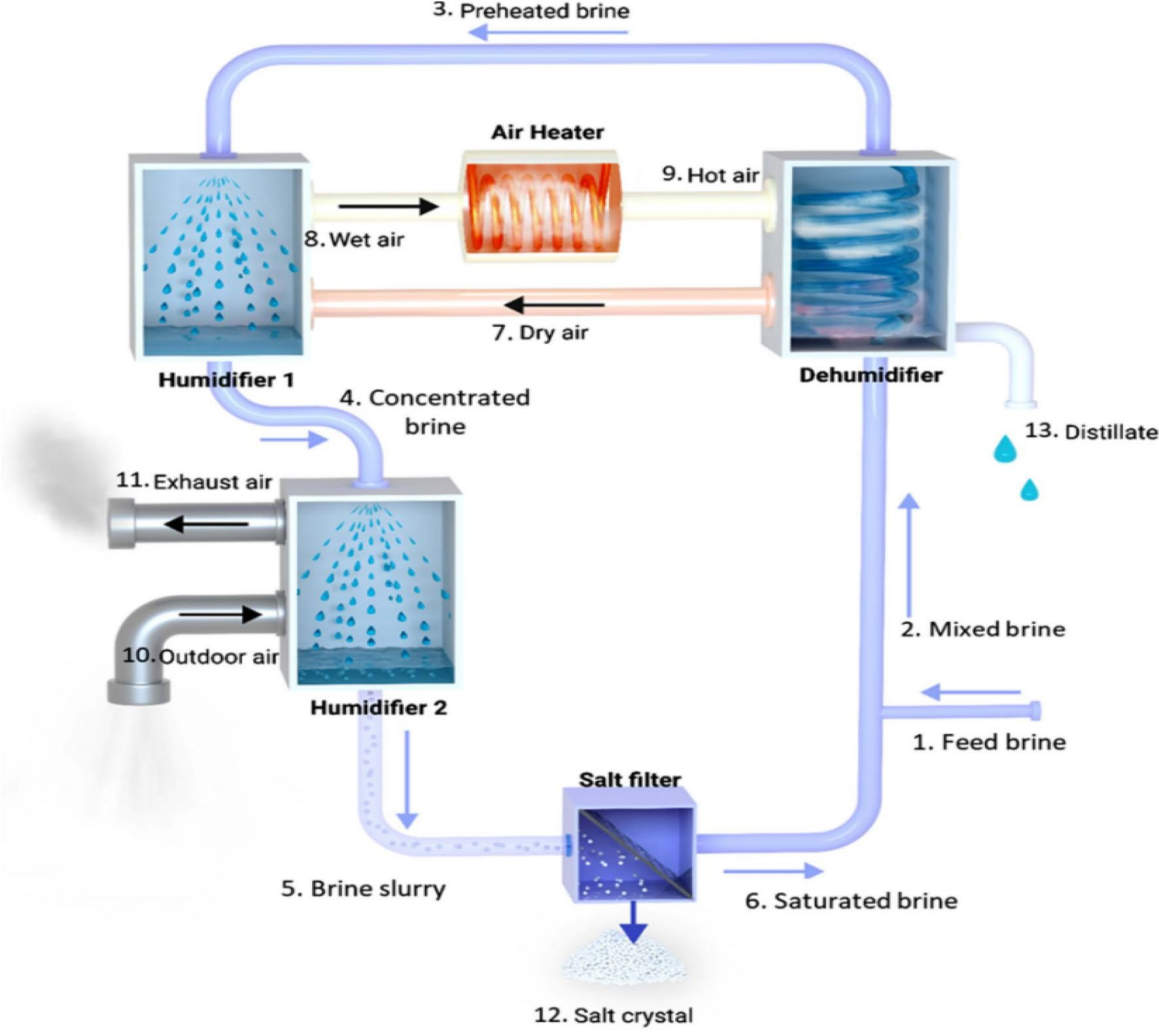
Schematic diagram of the crystallizer based on the HDH process (Chen et al. 2021)

Evaporative crystallization is the most effective brine treatment process because the main salt component in desalination brine, NaCl, has a flat solubility curve. In the literature, a number of evaporative crystallizers have been described. For the treatment of wastewater, Xu et al. (2010) introduced the multi-effect evaporation crystallization (MEEC) system. Three effects made up the system; the first two effects concentrated the brine, while the third effect served as a crystallizer. The liquid produced by this device had a concentration of less than 0.5 mg/L and produced salt that was 99.9% pure. An evaporative crystallizer combined with mechanical vapor recompression (MVRC) was examined by Han et al. (2009). Energy was conserved by compressing and reusing the steam created during evaporation as a heat source. Based on heat and mass balances, heat transfer calculations, and MVRC systems, Zhou et al. (2014) constructed a mathematical model. The model was able to produce results that were in good agreement with experimental data obtained from a laboratory plant with a 20 kg/h capacity. The particular compressor power might be decreased when the operating temperature difference was lowered, according to the analytical data. A two-effect MVRC system was created by Liang et al. (2013). Both energy consumption and operating expenses were significantly lower than in single-effect systems. Evaporative crystallizers are not frequently used in the desalination business despite intensive research efforts. The factors include a high initial plant cost, a complex design and operation, and a lack of experience with long-term operations (Lu et al. 2017). However, membrane distillation crystallization (MDC) has become a more viable option for brine treatment because of recent developments in MD. MD systems have a number of appealing characteristics, including as excellent salt rejection, low operating pressure and temperature, and high system compactness (Chen et al. 2020). These advantages should also apply to MDC systems, according to expectations. Therefore, during the past few years, MDC has attracted a lot of scientific interest. For the treatment of saturated brine, Edwie and Chung (2013) devised the simultaneous membrane distillation-crystallization (SMDC) system. A cooling crystallization module and a direct-contact membrane distillation (DCMD) module made up the system. When the feed temperature was raised from 40 to 70 °C, the rate of salt crystallization increased from 7.5 to 34 kg per m^3^ of feed, while the average crystallizer size reduced from 87.40 to 48.82 μm. In order to create a ZLD system, Lu et al. (2019) combined freeze desalination, DCMD, and freeze crystallization modules. The precise energy usage was estimated to range between 2000 and 2500 kWh/m^3^ under ideal operating circumstances. Nakoa et al. (2016) coupled solar ponds and DCMD for ZLD desalination. In order to increase the efficiency of the solar pond, the DCMD intake was fed from the non-convective zone while the discharge was directed to the lower convective zone. Fifty-two liter per square meter of membrane area was produced each day by the system. Guo et al. (2016) conducted an analytical analysis of a ZLD system that combines evaporative crystallization with air gap membrane distillation (AGMD). According to reports, the total energy consumption lies between 1700 and 2200 kJ/kg H_2_O. Aspen flowsheet simulation was used by Guan et al. (2012) to study an MDC system. The findings showed that crystallizer integration had a modest impact on overall energy usage because the MD heater provided the majority of the heat input. One of the main problems with MDC systems is their high scaling and fouling potential under high feed concentrations, which can result in a significant reduction in membrane flux (Julian et al. 2016) or even a total membrane blockage. As a result, membranes need to be cleaned and replaced frequently. This problem prevents MDC from being used more widely in brine treatment. Innovative salt crystallization methods are desperately needed to boost the ZLD’s viability. The humidification-dehumidification (HDH) cycle is compared to a unique crystallization technique in which air is used as a heat and mass transfer medium. Brine is concentrated by direct contact with hot, dry air to supersaturation levels. The desalination brine then crystallizes salt as vapor is transported to the condenser for the recovery of distillate and condensation heat. The advantages of this technique are low scaling and fouling potential, simplified component design, and lower heat input. Similar suggestions to use HDH for brine treatment have been made by Ghofrani and Moosavi (2020a, b). However, little emphasis was given to the crystallization process; instead, their investigations concentrated on the hybridization of HDH with other processes (such as MED and heat pumps) to reach higher thermodynamic and economic performances. An experimental setup to describe the evaporation and crystallization processes is developed in order to show the viability of using the HDH process for brine treatment. Mathematical modeling is then used to assess the HDH-based crystallizer’s thermodynamic performance. The advantages of the suggested technology are further demonstrated through a comparison to the evaporative crystallizer. The conclusions can be applied to the design and operation of innovative crystallization systems in the future. The high energy intensity and high plant costs of the crystallizers in existing ZLD systems constitute a limitation. The HDH process, which is the basis for the innovative crystallization method proposed in this study, has the advantages of low energy consumption, cheap component prices, and less scaling and fouling potential. First, a straightforward experimental setup is created to show that the suggested approach is workable. By using air that has been heated to 40 °C as the heat source, brine concentration and salt crystallization can be accomplished satisfactorily. After that, the entire system is subjected to a thermo-economic study. Per kilogram of feed brine, it is discovered that the specific thermal energy and power consumption levels vary between 700 and 900 and 5 and 11 kJ, respectively. The initial plant cost is decreased by 58%, and the energy consumption is 56% lower than that of a traditional evaporative crystallizer (Chen et al. 2021) (Fig. 4).

### Brine recycles HDH systems

Brine is frequently disposed of in the environment via a variety of techniques, including land application, deep-well injection, evaporation ponds, surface water discharge, and sewer discharge. Brine may contain hazardous pretreatment chemicals, organic substances, and heavy metals in addition to its extreme salinity. Numerous studies have looked into the harmful effects that brine disposal has on soil quality, groundwater quality, and the marine environment. Eutrophication, pH changes, an increase in heavy metals in marine settings, etc. are thus examples of potential environmental harm (Petersen et al. 2018). It is discovered that the brine recirculating approach can greatly lower the volume of rejected brine and is practically possible.

The most effective method of desalinating water is a ZLD configuration, which prevents discharge and does not produce any rejected brine. The brine is concentrated in ZLD systems almost to the point of saturation, where it crystallizes as salts. Evaporation-based techniques, such as sun evaporation ponds (Nakoa et al. 2016), or salt crystallizers can be used to crystallize the concentrated brine. The primary method for treating the rejected brine from desalination systems is evaporation, either naturally or artificially induced through the use of brine concentrators and wind-aided evaporation (Pereira et al. 2007). The elements affecting the features, effects, and management techniques of desalination brine are shown in Table 2.

**Table 2:** Brine characteristics, impacts, and management strategies

1	Factors influencing the desalination brine characteristics,	Climate and seasonal variations
		Water intake
		Desalination technique
		Discharge approach
2	Brine impacts	Change the marine water chemistry
		Alter Physiochemical composition of desalination brine
		Affect Marine water biodiversity(faun, flora and micro biome)
3	Management strategies	Zero liquid discharge
		Brine pretreatment

Akinaga et al. (2018) suggest using evaporative coolers in seawater greenhouses to minimize the brine volume, allowing for the growth of high-value products and the manufacturing of sea salt. However, using the suggested approach, sun evaporation ponds will be required to accomplish ZLD, but with a smaller area of the pond as the brine, the volume is greatly diminished. Mechanical vapor compression (MVC), membrane-based multi-stage methods, and multi-effect distillation (MED) have all demonstrated a high degree of water flux enhancement potential. As a low-energy method for brine volume minimization, integrated FO processes with RO, MSF, and MD have also been investigated. Membrane fouling, however, needs to be dealt with as a significant issue (Manzoor et al. 2017). Thermodynamic analysis of reversible, black box system components is conducted by Chung et al. (2017), while taking into account two distinct brine management techniques. The first technique combines salt production with total separation. Salinity-gradient power generation by pressure-retarded osmosis is the second brine management technique under consideration. A humidifier and a dehumidifier are the two heat and mass exchangers (HMEs) that make up a humidification-dehumidification (HDH) desalination system. Air in the humidifier comes into direct touch with saline water, but air in the dehumidifier might come into contact with water either directly or indirectly. The main three HDH types are described here: HDH with an indirect dehumidifier, HDH with direct contact, and HDH employing a bubble column. The use of air to pre-heat the saltwater in an HDH system with an indirect dehumidifier allows for more heat recovery. However, the saline water is only connected to the humidifier in direct-contact HDH systems. As a result, dehumidifier corrosion-related issues are resolved, and in this instance, the system’s capital investment cost is lower (Narayan et al. 2010). When it comes to reliability and affordability, HDH technology has been shown to be an environmentally friendly desalination method. For applications utilizing low-grade heat sources, it has been demonstrated that the HDH system is a cost-effective and dependable desalination system (Thiel et al. 2015; Dehghani et al. 2018). The capacity of several HDH system topologies to produce water was studied and evaluated by Narayan et al. (2010). They claimed that a multi-effect CAOW (close-air open-water) water-hated HDH system is more effective than previous arrangements. Numerous studies have evaluated the performance of HDH systems using direct contact dehumidifiers. A finite volume model is used to research and compare the effectiveness of co-current and counter-current dehumidifiers. In order to parametrically explore the performance characteristics of an open-air DC HDH system while taking into account various operational situations, Klausner et al. (2006) constructed a heat and mass transfer model. For a solar HDH system with a direct contact dehumidifier, Alnaimat and Klausner (2012) did a transient analysis to assess the desalination process under dynamic operating settings with transient variation during normal operation. A membrane distillation desalination system was used by Swaminathan to assess and rank several recirculation procedures, including batch, semi-batch, continuous, and multistage. Garg et al. (2018) created a mathematical model and investigated how well brine recirculation was working in multistage flash desalination. As a heat source, the system is connected to a solar collector that uses nanofluids for direct absorption. When using an indirect contact dehumidifier, Zubair et al. (2018) recirculated several brine flow rates to assess how well the HDH performed. In a single-effect MVC desalination system, Jamil and Zubair (2017) conducted a thermo-economic analysis with and without taking brine recirculation into account. According to their calculations, the MVC desalination system’s specific thermal energy consumption will be 13 kWh/m^3^ and 9.8 kWh/m^3^ with and without brine recirculation, respectively. Highly saline water supply can be used with HDH desalination systems. However, a major problem with brine management in HDH systems is the volume of rejected brine, which can have disastrous effects on the ecosystem. A brine recirculation method should be used by taking into account a bleeding flow of the concentrated brine stream in order to reduce the volume of rejected brine and raise the overall water recovery ratio in the HDH desalination system. As a result, the system may operate at steady-state high salinity feed (Dehghani et al. 2019).

## The technologies used in brine treatment

There are two types of technologies employed in brine treatment/ZLD systems: membrane-based and thermal-based technologies. The feed brine’s composition, the freshwater’s purity requirements, and the final concentration of the concentrated brine required for either safe disposal or other advantageous applications all have an impact on how a ZLD system is designed. 

### Membrane-based technology for brine treatment

Reverse osmosis (RO) is the most used membrane-based desalting technique for salty water. In the RO, the compartment with the greater salt concentration receives hydraulic pressure, which forces water molecules to pass through a semipermeable membrane in the compartment with the lower salt concentration. The feed brine’s (*f*) and the permeate liquid’s (*p*) different osmotic pressures must be balanced by the applied pressure gradient. As a result, the pure solvent (freshwater) is let to pass on the other side of the membrane while the solute (concentrated brine) is kept on the pressured side (Nagy 2019).

Forward osmosis (FO) is a membrane-based system that, in contrast to RO/HPRO, relies on osmotic pressure gradients instead of hydraulic pressure (Kumar et al. 2018).

Osmotically aided reverse osmosis is a new technology that was created as a result of recent advancements in the RO industry (OARO). The RO and FO philosophies are combined in the pressure-driven membrane-based technology known as OARO (Chung et al. 2017).

Membrane distillation (MD) is a membrane-based technology with a thermal drive. Its foundation is a vapor pressure gradient that can be created by the temperature difference across the hydrophobic microporous membrane. The membrane’s hydrophobic property inhibits liquid molecules from passing through the holes while allowing vapor molecules to do so. With a high rejection rate of over 99% and the ability to recover highly pure freshwater, separation is thus accomplished (Ashoor et al. 2016).

Membrane crystallization (MCr) is a portion of the MD that provides the opportunity to simultaneously obtain valuable solid crystal salts and freshwater. In the MCr, the distillate is in contact with the membrane on one side while the feed solution, which contains a non-volatile solution that is prone to crystallize, is in contact with the membrane on the other. Therefore, the difference in vapor pressure between the two portions causes the volatile substances, including water, to evaporate, be transported across the membrane, and then condense on the distillate side. This continues up until supersaturation is attained and crystal nucleation is initiated. The MCr system exhibits well-controlled nucleation and growth kinetics as a result of this characteristic, as well as greater crystallization rates (Profio et al. 2017).

### Thermal-based technologies for brine treatment

The most popular brine treatment methods in a ZLD system are brine concentrators and brine crystallizers.

The brine concentrator (BC) is primarily intended to be used as a vertical tube or falling film evaporator, but it can also be utilized as a horizontal spray-film and plate-type evaporator. The feed brine is delivered to a heat exchanger in the BC, which raises the temperature of the brine to boiling, before moving on to a deaerator, which eliminates non-condensable gases. The recirculating slurry is combined with brine after being added to the evaporator sump. As a result, the brine slurry is pumped to the concentrator’s top where it enters a network of heat transfer tubes. On the surface of the inner tube, where the flowing brine generates a thin coating, water evaporation takes place. Before inserting the vapor compressor, where further heat is injected, a portion of the brine evaporates and passes through mist eliminators. The heat from the compressor’s vapor is then transferred to the cooler brine that enters the evaporator tubes by moving to the exterior of the tubes’ walls. The heat from the compressed vapor is transmitted to the entering brine stream as it passes through the feed heat exchanger and condenses into freshwater. This technology’s SEC ranges from 15.86 to 26 kWh/m^3^ (Mickley 2008).

**Fig. 5:**
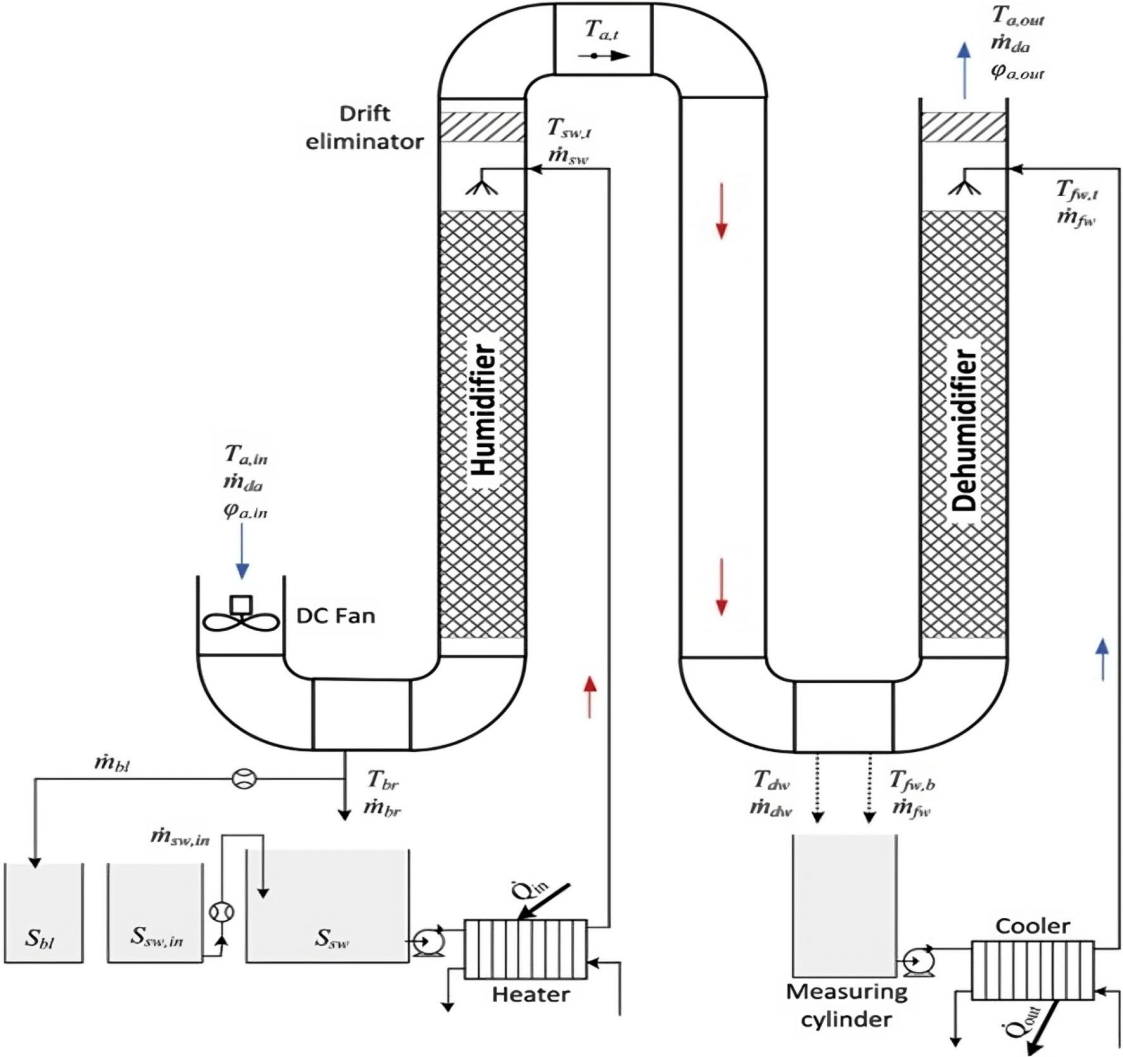
Schematic diagram of the HDH desalination system (Dehghani et al. 2019)

Brine crystallizers (BCrs) are generally built as vertical cylindrical vessels that receive heat from a steam source or vapor compressor that is readily available. The forced circulation crystallizer is the most used form of crystallizer for treating brine. The brine is originally put into the crystallizer sump in this method. In a shell-and-tube heat exchanger, the incoming brine is combined with the outgoing brine and heated by vapor from the vapor compressor. The brine is under pressure and does not evaporate since the heat exchanger’s tubes are submerged in water. Recirculating brine swirls in a vortex as it enters the crystallizer vapor body at an angle. A tiny amount of the brine evaporates and crystallizes. In order to extract the last of the water from the crystals, a small stream of the recirculating loop is passed to a centrifuge or filter while a substantial portion of the brine is returned to the heater. When the compressed vapor condenses on the heat exchanger, it heats the recirculating brine (Mickley 2008). Finally, a dry solid is created after collecting freshwater. Figure 5 shows a typical schematic representation of BCr (b). Although BCr can be used directly on brine, its capital cost and energy requirement are significantly higher than those of an equivalent-capacity BC. However, BCr’s key benefit is that brine up to 300,000 mg/L TDS can be utilized with it. This technology’s SEC ranges from 52 to 70 kWh/m^3^. For every cubic meter of freshwater produced, BC costs about US$1.11 while BCr costs about US$1.22 (Stanford et al. 2010).

### Evaluation and comparison of desalination brine treatment technologies

Based on how well they can handle the desalination brine, these technologies could be rated regarding a few factors and indicators, such as energy usage, general performance, environmental effects, difficulties, and hopes for the future.

### Energy consumption

ZLD systems’ high energy consumption is a serious issue that prevents further adoption as well as the development of thermal and membrane-based technologies (Panagopoulos et al. 2019; Alnouri et al. 2017). Technologies based on membranes prevent energy losses brought on by condensation and evaporation. Thermally driven membrane-based technologies have a substantially greater energy demand (39–73 kWh/m^3^) than membrane-based technologies with an SEC that ranges from 0.6 to 19 kWh/m^3^ (Wenten et al. 2017).

### Overall performance

It is important to take into account the water recovery and the price of the produced freshwater. RO is the most popular desalination method, but due to salinity restrictions (b70,000 mg/L TDS) and poor recovery, RO is hardly ever utilized in brine treatment (b50%). As a result, RO is frequently utilized to desalinate BW/SW first, with more efficient technologies in the ZLD systems coming after (Mickley 2008). Despite having great performance (b99% recovery), the commercial ZLD technologies BC and BCr have such high capital and operating costs that other options are being examined. For instance, MSF/MED might be an excellent substitute because of how little energy they use. However, there is still a significant risk of scaling in the heat exchangers.

### Environmental impacts, challenges, and prospects

ZLD’s implementation can have unintended negative effects on the environment, even though its main goals are to maximize freshwater recovery and reduce waste. The resulting mixed solid salts cannot be utilized again and, when kept in evaporation ponds, emit unpleasant smells, threaten nearby wildlife, and potentially represent a leaking risk. In order to prevent potential contamination from solid waste, impermeable liners and reliable monitoring systems are needed for two reasons: (i) to minimize the requirement for and costs associated with solid waste disposal and (ii) to sell the high-purity salts and adopt a circular economy strategy. Today, a fast-growing technique is to manufacture many high-purity solid salts instead of a compact mixed solid salt. Salts like NaCl, CaCO_3_, Na_2_SO_4_, and CaCl_2_ are of interest in this method. In light of the properties of the feed brine solution, several ZLD systems can be created to recover both freshwater and high-purity solid salts (Stanford et al. 2010).

## Impact of salinity on the total cost

The primary goal of the cost analysis for the suggested system is to examine the system’s viability in terms of the upfront costs and the costs associated with producing freshwater. The cost of the supply well; equipment costs such as pipelines, tanks, and pumps; and land and building costs, if indoor space is needed, are all included in the capital cost of a conventional desalination plant. The prices could also cover things like shipment, building, and services.

Thus, the equation below might be used to determine the cost of producing freshwater;

$$\frac{\mathrm{Total capital cost}}{\mathrm{Annual freshwater total }\times \mathrm{system lifetime}}=\mathrm{cost per liter }\left(\frac{\mathrm{\$}}{\mathrm{L}}\right) (\mathrm{Zubair et al}., 2017)$$

The cost of desalination is significantly influenced by a wide range of parameters. A good selection of parts (materials and equipment) with high efficiency and the best quality/cost ratio, the use of energy recovery and energy storage systems, the design of the system adapted to the unique local conditions, and the use of appropriate maintenance schedules are all potential cost-reduction strategies. The price of desalinating water is considerably impacted by increased salinity. Energy use typically increases with salinity (Zubair et al. 2017). Salinity impacts can be seen in the computation of energy consumption in two different ways: the salty streams’ decreased specific heat and the humidifier’s decreased vapor pressure (Thiel et al. 2015).

The largest water output of the HDH desalination system is found to be inconsistent with the situation with the lowest capital costs. With improving efficiency for both humidification and dehumidification, the overall cost for the heat and mass transfer area would rise (He et al. 2018).

## Key challenges and future consideration

There are other system design and operational variable techniques that require more research, such as the following:

- Challenges in HDH recommend conducting more experimental investigations on HDH driven by renewable energy that have not been tried on a large scale.
- Integrating renewable and alternative energy technologies to improve some technical and economic aspects, such as the production rate and gained output ratio, is a strong step towards sustainability. This also lowers overall costs and lessens the environmental impact of desalination, leading to the long-term production of efficient systems.
- Simulate every system component with various designs, sizes, insulators, and packaging to effectively cut down on investment and operational expenses.
- Energy recovery in systems is crucial and demands further attention to improve the performance of HDH systems.
- A water desalination system may be commercially viable if the heat waste from industrial operations or power plants is used as a heat source.
- It is difficult to reduce the amount of rejected brine and raise the overall water recovery ratio in the humidification-dehumidification system since this problem has serious environmental consequences.
- Based on the properties of the feed brine solution, various ZLD systems can be built to recover both freshwater and high-purity solid salts.

## Conclusions

The HDH desalination technology can work with a variety of salinities. However, increased salinity has an impact on system productivity. The productivity of water per unit of time decreased significantly as the mass fraction of salt increased, but the quality of pure water did not change. Instead, the specific thermal energy consumption increased noticeably.

Here are some additional details.

- The HDH is regarded as one of the best desalination methods for handling the serious problem of drinking water scarcity as it can handle very salinized water that other desalination technologies cannot handle.
- Based on the energy consumed, cycle configuration, and heating system, the HDH WDS may be divided into three basic categories:
- Due to its low operating temperature, the HDH system can be powered by renewable energy sources including solar and geothermal energy.
- The management of brine is growing in importance in the water processing sector. Other disposal techniques, such as brine discharge into open water bodies, are not always feasible and environmentally unsustainable. The need for brine concentration has grown as a result of rising legislation and environmental concerns.
- ZLD systems’ dual goal of recovering freshwater and solid salts, which prevents wastewater discharge in the environment, makes them an attractive option to disposal techniques. Resource recovery may serve as an additional economic driver for the development of ZLD technology in addition to water recycling.
- In terms of the amount of water generated, the particular renewable energy needed, and the specific cost of water produced, more changes are required to maximize process performance metrics.

## Recommendation

- Salinity has a substantial impact on how well the HDH system performs. In some circumstances, increasing energy efficiency can be achieved by controlling the salinity of the HDH system.
- ZLD can have an influence on the environment even though its main goals are to maximize freshwater recovery and reduce waste. The created mixed solid salts cannot be recycled and pose a leakage risk. To avoid paying for solid waste disposal and to employ high-purity salts, it is now necessary to generate high-purity solid salts rather than mixed solid salts.

## Nomenclature

ABHP: Absorption heat pump
AD: Adsorption desalination
AGMD: Air gap membrane distillation
AH: Air heating
BC: Brine concentrator
CAOW: Closed air open water
CPVT: Concentrated photovoltaic-thermal
CWOA: Closed water open air
DCMD: Direct-contact membrane distillation
DEAB: Double-effect absorption
ECS: Ejector cooling system
MD: FO
FPSWH: Flat plate solar water heater
HDH: Humidification-dehumidification
HFUA: High-frequency ultrasonic atomizer
HP: Heat pump
HSHDH: Hybrid solar humidification-dehumidification
KC: Kalina cycle
MCr: Membrane crystallization
MD: Membrane distillation
MDC: Membrane distillation crystallization
MED: Multi-effect distillation
MEEC: Multi-effect evaporation crystallization
MS: Multi-stage
MSF: Multi-stage flash
MTBC: Multi-tray bubble column
MVC: Mechanical vapour compression
MVRC: Mechanical vapour recompression system
OAOW: Open air open water
OARO: Osmotically aided reverse osmosis
PV: Photovoltaic
PVT: Photovoltaic-thermal
RO: Reverse osmosis
SGSP: Salinity-gradient solar pond
SMDC: Simultaneous membrane distillation-crystallization
SSF: Single stage flashing
SWRO: Seawater reverse osmosis systems
TDS: Total dissolved solids
TEAB: Triple-effect absorption
VHDH: Vacuum humidification-dehumidification
WDS: Water desalination system
WH: Water heating
ZLD: Zero-liquid discharge

## Symbol

*Cp*: Specific heat capacity at constant pressure (kJ/kg K)
*F*: Energy reuse factor
*GOR*: Gain output ratio (–)
*ṁ*_*fw*_: Mass flow rate of freshwater
*MR*: Mass flow rate ratio
*ṁ*_*sw*_: Mass flow rate of feed seawater
*Q̇*_*in*_: Total energy input
*RR*: Recovery ratio (–)
*S*: Salinity (%)
*S*_*gen*_: Specific entropy generation
*T*: Temperature (°C)

## Greek letters

*λ*: Latent heat of vaporization
